# Supplementary material for: An Easy and Ecological Method of Obtaining Hydrated and Non-Crystalline WO3−x for Application in Supercapacitors
Source: Materials (Basel). 2020 Apr 19;13(8):1925. doi: 10.3390/ma13081925 (PMC7215928; doi:10.3390/ma13081925)
Supplement: Supplementary file 1 [file materials-13-01925-s001.pdf]

materials

MDPI

Article

Supplementary Materials: An Easy and Ecological Method of Obtaining Hydrated and Non-Crystalline WO_{3-x} for Application in Supercapacitors

Mariusz Szkoda [1,*], Zuzanna Zarach [1], Konrad Trzciński [1], Grzegorz Trykowski [2] and Andrzej Nowak [1]

1 Faculty of Chemistry, Department of Chemistry and Technology of Functional Materials, Gdańsk University of Technology, Narutowicza 11/12, 80–233 Gdańsk, Poland; zuziaz696@gmail.com (Z.Z.); trzcinskikonrad@gmail.com (K.T.); andnowak@pg.edu.pl (A.N.)

2 Faculty of Chemistry, Nicolaus Copernicus University in Toruń, Gagarina 7, 87–100 Toruń, Poland;; tryki@umk.pl (G.T.);

* Correspondence: mariusz.szkoda@pg.edu.pl

Received: 22 March 2020; Accepted: 16 April 2020; Published: date

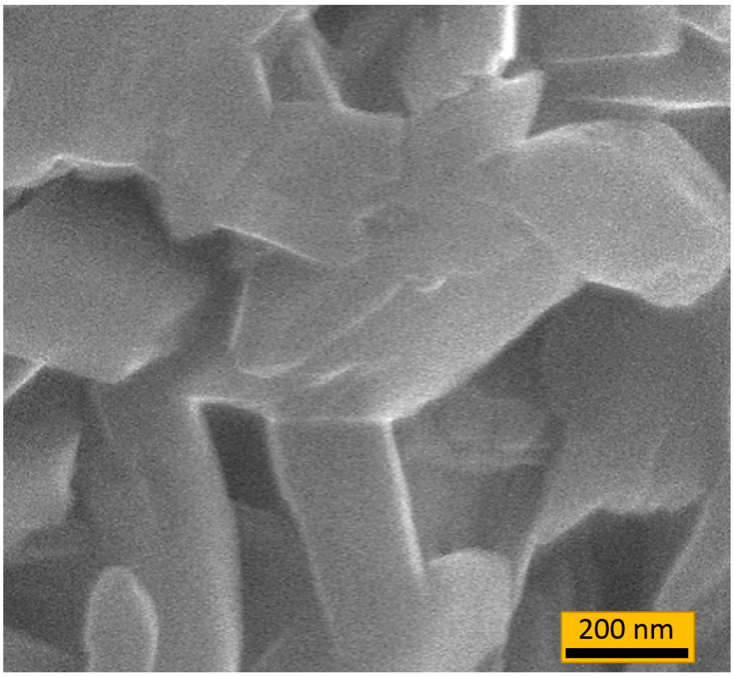

Figure S1. SEM on higher magnification for hydrated WO_{3-x}.

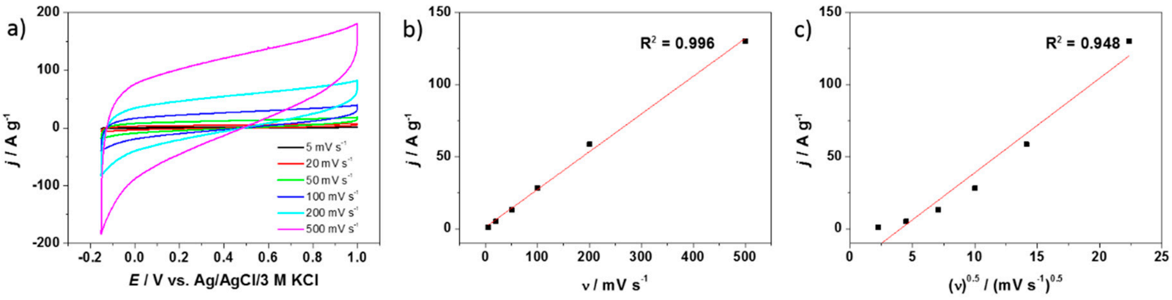

Figure S2. (**a**) CV curves of hydrated WO_{3-x} electrode in 0.2 M K_2SO_4. Scan rates 5–500 mV s^{-1}. Dependence of anodic current at 0.5 V (**b**) vs. scan rate and (**c**) vs. square root of the scan rate.

Three of four models were fitted to data using the same equivalent circuit that consists: R1 – electrolyte resistance, R2 – resistance on the electrode/electrolyte interface, R3 – bulk resistance, CPE1 – constant phase element on the electrode/electrolyte interface, and CPE2 – constant phase element in bulk (EQC 1). In the case of the spectrum of bulk WO_3 recorded at a rest potential, additional Warburg element has to be added in order to fit model properly (EQC 2).

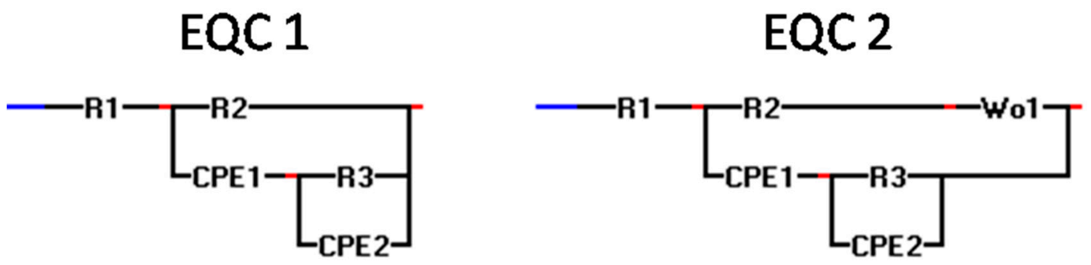

Figure S3. The equivalent circuits used for modeling.

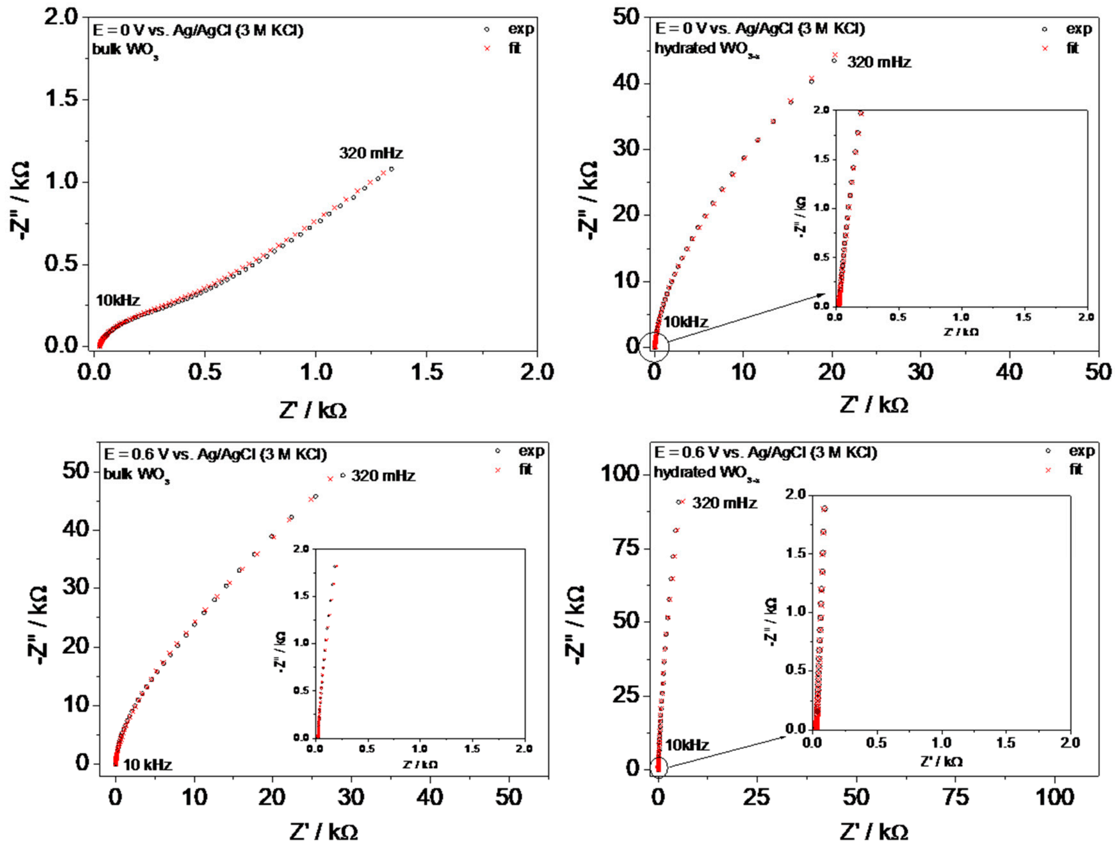

Figure S4. The impedance spectra, experimental and fitted, of bulk WO_3 and hydrated WO_{3-x} recorded at 0 V and 0.6 V vs. Ag/AgCl (3 M KCl).

In the case of measurements performed under 0.6 V, the spectra of hydrated WO_{3-x} exhibits typical capacitive behavior. The most significant differences of fitting parameters between electrodes under anodic polarization are seen in the values of R2, R3, and P2 (from CPE2). It can be concluded that resistance on the electrode/electrolyte interface is higher for modified WO_3, however, the resistance within electrode material is 4 order of magnitude lower for hydrated WO_{3-x}. The P2 parameter of CPE2 (in the case of n close to 1) mainly contains a capacitive component. The one order of magnitude higher value was obtained for material after proposed modification. Thus, EIS analysis confirms that hydrated WO_{3-x} material can act as an electrode material for supercapacitors.

Table S1. The results of fitting procedure.

	At 0.6 V		At 0 V	
	**Bulk**	**Hydrated WO_{3-x}**	**Bulk**	**Hydrated WO_{3-x}**
R1 / Ω	24.00	25.72	23.15	26.01
R2 / Ω	1.54E+05	3.60E+06	336.83	1.70E+05
R3 / Ω	1.08E+04	4.66	254.54	3966.50
P1 / $\Omega^{-1}s^n$	8.05E-06	5.54E-06	1.63E-05	9.99E-06
n1	0.99	0.98	1	0.95
P2/ $\Omega^{-1}s^n$	1.55E-05	1.39E-04	0.00011	4.38E-05
n2	0.93	0.83	0.77	0.99
W_{or} / $\Omega s^{-0.5}$	-	-	1466.40	-
W_{oc} / $s^{0.5}$	-	-	7.92	-

Table S2. Comparison of the electrochemical properties of the non-crystalline WO_3 with some previous reports on metals oxide-based supercapacitors.

Electrode material	C_s [F g^{-1}]	Current density or scan rate	Energy Density [Wh kg^{-1}]	Power Density [W kg^{-1}]	Ref.
NiO nanoflakes/rGO	50	1 mA/cm^2	39.9	-	[32]
MnO_2/Fe_2O_3 nanorods	89	0.5 mA/cm^2	0.41 mWh/cm^3 (at 0.5 mA/cm^2)	0.1 W/cm^3 (at 6 mA/cm^2)	[33]
MnO_2 nanorods	449	0.75 mA/cm^2	0.25 mWh/cm^3 (at 2 mA/cm^2)	1.44 W/cm^3 (at 12 mA/cm^2)	[35]
$CuCo_2O_4$@NiO	124.6	1 A/g	38.9	750	[66]
Co_3O_4-rGO/Ni	80	0.1 A/g	20	1200	[67]
Hexagonal WO_3	484	0.93 A/g	25	89	[68]
Graphene-TiO_2	165	5 mV/s	12.5	1440	[69]
MnO_2-RuO_2@GNR	156	1 A/g	60	14000	[70]
$Cu_2O/CuMoO_4$ nanosheets	156	1 A/g	75.1	420	[71]
Pd doped monoclinic WO_3	41	0.5 A/g	10.6	198	[72]
Graphene-WO_3 Nanowires	465	1 A/g	26.7	6000	[73]
WO_3-MnO_2	103	5 mV/s	24.13	915	[74]
Hydrated WO_{3-x}	122	0.5 A/g	60	803	This work
